# What is the impact of psychiatric decision units on mental health crisis care pathways? Protocol for an interrupted time series analysis with a synthetic control study

**DOI:** 10.1186/s12888-020-02581-5

**Published:** 2020-04-23

**Authors:** L. P. Goldsmith, J. G. Smith, G. Clarke, K. Anderson, J. Lomani, K. Turner, S. Gillard

**Affiliations:** 1 Population Health Research Institute, St George’s, University of London, Cramner Road, Tooting, London, SW17 0RE UK; 2 The Health Foundation, 8 Salisbury Square, London, UK

**Keywords:** Interrupted time series, Synthetic control, Mental health crisis, Service pathways, Service parameters, Psychiatric decision unit, emergency department, crisis management, inpatient alternatives, psychiatric crisis

## Abstract

**Background:**

The UK mental health system is stretched to breaking point. Individuals presenting with mental health problems wait longer at the ED than those presenting with physical concerns and finding a bed when needed is difficult – 91% of psychiatric wards are operating at above the recommended occupancy rate. To address the pressure, a new type of facility – psychiatric decision units (also known as mental health decision units) – have been introduced in some areas. These are short-stay facilities, available upon referral, targeted to help individuals who may be able to avoid an inpatient admission or lengthy ED visit. To advance knowledge about the effectiveness of this service for this purpose, we will examine the effect of the service on the mental health crisis care pathway over a 4-year time period; the 2 years proceeding and following the introduction of the service. We use aggregate service level data of key indicators of the performance of this pathway.

**Methods:**

Data from four mental health Trusts in England will be analysed using an interrupted time series (ITS) design with the primary outcomes of the rate of (i) ED psychiatric presentations and (ii) voluntary admissions to mental health wards. This will be supplemented with a synthetic control study with the same primary outcomes, in which a comparable control group is generated for each outcome using a donor pool of suitable National Health Service Trusts in England. The methods are well suited to an evaluation of an intervention at a service delivery level targeting population-level health outcome and the randomisation or ‘trialability’ of the intervention is limited. The synthetic control study controls for national trends over time, increasing our confidence in the results. The study has been designed and will be carried out with the involvement of service users and carers.

**Discussion:**

This will be the first formal evaluation of psychiatric decision units in England. The analysis will provide estimates of the effect of the decision units on a number of important service use indicators, providing much-needed information for those designing service pathways.

**Trial registration:**

primary registry: isrctn.com Identifying number: ISRCTN77588384 Link: Date of registration in primary registry: 27/02/2020.

**Primary sponsor:**

St George’s, University of London, Cramner Road, Tooting, SW17 ORE. Primary contact: Joe Montebello.

## Background

Mental health crisis care is under extreme pressure internationally [1–5]. In the UK, bed occupancy is high, staff are under pressure and resources are constrained – the UK Emergency Department (ED; also known as Accident and Emergency) system has been described as near breaking point [6]. In the UK, two-thirds of people attending the ED multiple times for any reason have had previous contact with specialist mental health services or have previously been admitted to an acute hospital for a mental health condition [7], with frequent attenders at greater risk of psychiatric inpatient admissions [8]. People presenting with a mental health issue are over 6 times more likely than people presenting with a physical concern to wait more than 4 hours at the ED [9]. Bed occupancy in inpatient psychiatric facilities is well above recommended levels with 91% of wards operating above the recommended occupancy rate [10]. Mental disorders are estimated to account for around 5% of ED attendances in the UK and almost 30% of acute inpatient bed occupancy and acute readmissions [7, 9, 10]. Although the emergence of liaison psychiatry services has enabled organisations to provide responsive mental health advice and assessment within emergency care settings, there remain wide variations in service provision [11] and ongoing challenges to sustainability [12]. The introduction of crisis resolution and home treatment teams and triage wards has offered little benefit in reducing contact with acute services, inpatient admissions or costs across the wider in-patient system compared with standard models of care [13–15], with ongoing staff concerns over the accuracy of triage decisions for mental health presentations [16, 17], while inpatient care remains unpopular, expensive and sometimes detrimental for individuals and their families [7, 18–20].

Against this context, Psychiatric Decision Units (PDUs; also called Mental Health Decision Units, and sometimes called Assessment Suites) have been introduced into the crisis care pathway in some National Health Service trusts in England [18, 21] (Crowe C, Walsh D: Lotus assessment suite PDU (psychiatric decision unit) - nine-month performance report: Springfield University Hospital: South West London and St George's Mental Health Trust, unpublished). PDUs are designed to reduce admissions to acute inpatient care, especially avoidable short admissions and expensive out of area or private admissions, reduce subsequent crisis presentations at the ED, and are an integrated part of the crisis care pathway [18] (Creswell J, Beavan M: Accreditation for inpatient mental health services (AIMS) standards for assessment/triage wards. London: Royal College of Psychiatrists, unpublished). These are short-stay facilities, based within psychiatric hospitals, for referred voluntary patients in acute mental health crisis who receive time-limited help (typically up to 24–72 h), after which discharge to the community or an inpatient admission occurs. The targeted referrals of people who experience excessive stays in emergency departments, frequent use of other services, such as the police and ambulance services, and who have complex and frequent crisis-related needs, distinguishes PDUs from triage or assessment wards, which accept all patients requiring assessment or treatment from the crisis care pathway and have a short length of stay (Creswell J, Beavan M: Accreditation for inpatient mental health services (AIMS) standards for assessment/triage wards. London: Royal College of Psychiatrists, unpublished). Additionally, PDUs typically only accept voluntary patients – whereas Triage Wards will admit people under assessment or treatment sections of the Mental Health Act, and the focus on PDUs is about assessment and planning on community-based provision in the medium term rather than inpatient care. The units are often nurse-led with consulting input from psychiatry and other mental health professionals. The 24 h facilities offer enhanced mental health assessment and short-term support which is targeted for people for whom inpatient admission is being considered (differing in function from the triage wards found in some mental health trusts which admit all people requiring inpatient care for purposes of assessment for ongoing care) (Creswell J, Beavan M: Accreditation for inpatient mental health services (AIMS) standards for assessment/triage wards. London: Royal College of Psychiatrists, unpublished).

International counterparts to the PDU are often known as a Psychiatric Emergency Service (PES) or Crisis Stabilisation Unit (CSU), and have become increasingly critical to the delivery of mental health crisis care [22–24]. This is particularly true for the US where a third of hospitals are estimated to provide these emergency units [25, 26], but they are also present in France [27] and Singapore [28]. Although formal evaluations of recently developed (single site) PDUs in the US and Australia have suggested these type of units might reduce length of stay in emergency departments and inpatient psychiatric admissions among patients with mental health presentations accessing emergency care [29, 30], evidence regarding the characteristics of effective and acceptable PDUs in England is restricted to informal local evaluations [21, 31] (Crowe C, Walsh D: Lotus assessment suite PDU (psychiatric decision unit) - nine-month performance report: Springfield University Hospital: South West London and St George's Mental Health Trust, unpublished). While these reports suggest the service model has potential to reduce demand on the ED, key data have not been reported (e.g. length of stay in the ED).

It is possible PDUs introduce further fragmentation to the system, and, if not effective, may waste critical resources. As such, a formal evaluation of these services is urgently required to describe the model of care and generate much needed knowledge about the impacts, quality, and cost benefits of a new assessment-based service for people experiencing mental health crises and accessing emergency services. The study aims to evaluate the effectiveness of decision units by examining the effect on key indicators of performance of the mental health crisis pathway.

The proposed work, (Evaluating Mental Health Decision Units in acute care pathways (DECISION): A quasi-experimental and health economic evaluation), will be the first formal evaluation of PDU services in England and the only project to date that includes comparison of different PDUs.

### Study setting

This research takes place in four geographical locations in England, each of which have an PDU. To maximize generalisability, sites have been chosen where there is variation in the configuration of the PDUs across the four sites to allow comparison and ascertain the optimal PDU configuration (see Table 1).

**Table 1:** Structural variation in Psychiatric Decision Units

Trust with PDU	Maximum Length of Stay	Capacity	Referral route
Inner city 1	48 h	5	ED (liaison), Crisis Resolution and Home Treatment Teams, Street Triage
Inner city 2	24 h	10	ED (liaison), Out of Hours Team, Approved mental health professionals team, Mental health inpatient wards, 136 Place of Safety, Community teams, including crisis resolution and home treatment teams
Inner city 3	72 h	8	ED (liaison), Street Triage
Rural Site	24 h	6	ED (liaison), Crisis Resolution and Home Treatment Teams

Referrals to PDU can be made from a range of services including ED liaison psychiatry teams, Crisis Resolution & Home Treatment teams and street triage teams. Referrals are triaged at a decision point prior to admission to the PDU. Overnight accommodation is single sex and units tend to be small, with a staff: service user ratio of at least 1:2 to allow substantial time for detailed assessment, brief intervention and onward referral/ signposting. Therapeutic work is typically cognitive or psychosocial, for example, improving resilience and solution focused approaches. Pathways from the decision unit can be either subsequent admission, discharge or forward signposting to appropriate recovery and preventative services.

### Aims

The aim of the study is to identify the impact of psychiatric decision units on mental health crisis care pathways. We will do this by administering an interrupted time series, supplemented by a synthetic control study, using routinely collected mental health trust and emergency department data. We will produce estimates of the extent to which changes in key service parameters (e.g., informal psychiatric admissions and mental health presentations at the ED) in the pre- and post-interruption period are explained by the introduction of PDUs at each of the sites in the study, and combine the results across sites to give a generalizable effect size estimate.

## Methods

### Study design

We will use an interrupted time series design, supplemented by a synthetic control study [32]. Adults (over 18) in psychiatric crisis in England of both sexes are eligible. The data collection periods started in November 2012 and will end on March 31st 2020. This project has been given favourable opinion from the East Midlands Leicester South Research Ethics Committee (19/EM/0226) and entry to the study is currently open. The research is funded by the National Institute of Health Research (NIHR) Health Services and Delivery Research (17/49/70). The current protocol described is version 4.0, dated 16th December 2019, and relevant parties will be informed of any important protocol modifications. The research uses aggregate service use data, and so Trusts, rather than individuals join the study. A data monitoring and ethics committee is not needed as there is no experimental intervention. The Independent Steering Committee (which includes an academic chair, a statistician, and two public and patient members) provides oversight and advice.

An interrupted time series (ITS) design, using routinely collected healthcare data, will explore change in acute and psychiatric hospital activity after the introduction of PDUs in our 4 sites, hereafter referred to as the treated trusts. Quasi-experimental methods, such as an ITS design, are appropriate when the randomisation and/or the ‘trialability’ of the intervention is limited. They are particularly well suited to evaluations of organisational interventions or changes to health care at a delivery level that target population-level health outcomes and when a time series is available, as in this instance [33, 34]. In ITS studies, data are collected at multiple time points before and after the introduction of a change or intervention; enabling detection of whether or not the change has a significantly greater effect on outcomes of interest than any underlying secular trend [35]. ITS findings primarily concern whether the level and/or slope of the outcome measurement is altered once change has been implemented. Advantages of the method include controlling for baseline variation, periodicity, cyclical trend and/or autocorrelation in the time series design, prior to examination of change effects. The number of observations is important; examining a long series of outcome measurements more readily allows analyses to track both immediate and delayed effects [36].

A synthetic control study in which comparative control trusts (in which no change in service delivery/ model of care occurs) are included in an interrupted time series analysis, improves the specificity of the evaluation and better controls for secular trends over the baseline, change or intervention, and follow-up periods [34]. Advanced synthetic control methods [37] will be used for controlled analysis to estimate a robust counterfactual against which to compare the impact of the introduction of each of the four participating PDUs on outcome variables. A mapping study will be used to identify suitable control trusts for the synthetic control study (sites without a PDU). A timeline of changes to the crisis care pathway will be elicited through interview with key informants and will be used to interpret the analyses.

### Sample size: ITS

The weekly series for 24 months pre- and post-implementation of PDUs (208 weekly or 48 monthly time points) is more than the 40 data points (20 pre- and 20 post-change) typically considered as adequate for valid ITS model analysis [38], and provides sufficient power to detect medium effects where they exist. For example, to detect a time x slope interaction with medium effect size on an outcome, a sample of 208 time points has > 99% power (assuming one parameter tested and no more than five factors entered in model; calculated using G-Power, ‘linear multiple regression: fixed model, r^2^ increase’ module).

### Sample size: synthetic control study

The monthly series for 24 months pre- and 24 months post-implementation of PDUs (including a one-month ‘bedding in month’), yields 49 time points. This provides us more than the 40 data points (20 pre- and 20 post-change) typically considered as adequate for valid ITS model analysis [38], and provides sufficient power to detect medium effects where they exist.

### Data collection

#### Interrupted time series

Outcome data will be collated as time series for 24 months pre- and 24 months post-implementation of the PDU. All data will be aggregated to a single observation at multiple time points at equal intervals. In our case, depending on the variable under study, the time series aggregation unit will be weeks or months. For out of area admissions, for example, a weekly total may be too low to appropriately use a weekly time series as differences may reflect random variation rather than a real effect (i.e. a high noise-signal ratio). The aggregation unit used will be the one that provides proportionately better model fits. Aggregated service use data over the relevant 4 years will be sourced locally from mental health trusts and the ED (acute hospital trusts) of participating PDU sites, as detailed above, through contact with Information Management & Technology (IM&T) departments at each trust. Where a substantial proportion of PDU patients (≥ 25%) are referred from a second acute trust, we will use data from that trust also. All data is handled in accordance with the UK Data Protection Act 2018 (incorporating the EU20 General Data Protection Regulation). Information Management Services personnel within the NHS Trusts will collate the trustwide service use data required for study analyses. Electronic transfer of this data between NHS Trusts and the lead university (St George’s, University of London [SGUL]) will be by encrypted email transfer. This data does not include personally identifiable information and will not be shared by SGUL with any third party or linked with other data that might render the information more identifiable. All data held on NHS or university computers will be securely held on password protected servers and not on individual PCs. The datasets generated during and/or analysed during the current study will be stored in a non-publicly available repository.

### Primary and secondary outcome measures

The primary and secondary outcome measures are shown in Table 2. We have a number of secondary outcomes as PDUs are understood by trusts to have wide-ranging effects on the crisis care pathway, and this approach allows each of these claims to be investigated. In Table 2, we also specify the denominator used to obtain the rate. A number of different methods have been used to calculate hospital catchment populations (HCPs) in England [39]. Here we will use the proportionate flow methods used by Public Health England (PHE) [40, 41].

**Table 2:** Outcome measures

**Primary outcome measures: Synthetic Control (ITS) Study**	
Rate of admissions to mental health trust adult inpatient wards per 1000 trust catchment population	Rate of ED psychiatric presentations per 1000 trust catchment population)
**Primary outcome measures: ITS Study**	
Rate of informal admissions to mental health trust adult inpatient wards per 1000 trust catchment population	Rate of ED psychiatric presentations per 1000 trust catchment population)
**Secondary outcome measures (both studies)**	
**Mental health trust**	**Acute trust**
Rate of inpatient admissions per 1000 trust catchment population	Proportion of 4-h psychiatric ED breaches (denominator psychiatric ED presentations)
Proportion of 0–5 day inpatient admissions (denominator total inpatient admissions)	Average length of psychiatric ED wait
Average length of inpatient stay (bed days)	Proportion of psychiatric ED 12 h trolley waits (denominator psychiatric ED presentations)
Proportion of compulsory admissions (denominator total admissions)	Proportion of psychiatric ED admissions admitted to an acute bed (denominator psychiatric ED presentations)
Proportion of psychiatric liaison episodes at the ED (denominator psychiatric ED presentations)	Proportion of psychiatric ED arrivals by ambulance or police (denominator psychiatric ED presentations)
**ITS only outcomes:**	
Daily mean occupied bed days (denominator size of trust catchment population)	
Proportion of out of area admissions (from the site MH trust to other MH trust or private provider) (denominator total inpatient admissions)	

An additional analysis for all mental health trust outcomes will be conducted considering only people with discharge from psychiatric inpatient services in the preceding 24 months. In a similar manner, additional analyses of the ED psychiatric presentations will be administered considering only people with a previous ED visit in the last 24 months. This is to ensure that we can estimate the effect of introducing PDU on services with respect to those populations most likely to be repeat users of mental health inpatient care and the ED, respectively.

#### Service mapping

We will map the PDUs in England to identify suitable trusts to be used as controls in the synthetic control analysis. We want to ensure that control trusts do not also have PDUs as this will dilute our ability to detect impacts in the treated trusts. Freedom of information (FOI) requests will be sent to all mental health trusts in England to identify the prevalence of PDUs in England. We will use trust websites to generate a contact list and send them the FOI request in the form of an electronic survey.

We will identify the scope of PDUs, the provision of care offered and variation in unit configuration to identify variations in service provision and aid dissemination of the results of the study. In addition, we will obtain information from each trust about alternative assessment and crisis care provision, including assessment wards; community-based assessment services and crisis services more generally, such as crisis houses and street triage teams. This will enable us to contextualise our findings when establishing the current cost and clinical benefits of PDUs in England as well as modelling the cost and impact of widespread roll out of PDU provision.

#### Synthetic control study

For the synthetic control study, aggregated service use data over the relevant 49 months for both the treated trusts and the selected control trusts will be sourced from Hospital Episodes Statistics (HES) and the Mental Health Services Data Set (MHSDS). The relevant time period is 49 months, as the month following the introduction of the PDU is excluded from the analysis to allow a bedding in time for set-up. HES and MHSDS are databases recording all admissions, outpatient appointments and ED attendances at NHS hospitals in England and mental health NHS trust service use respectively (NHS Digital, [42]).

#### Key informant interviews

Semi-structured interviews (*n* = 5 per site) will be held with strategic managers in each site, including PDU manager, Acute Care Pathway lead, mental health lead commissioner (or their equivalent locally) and the ED manager and ED clinical director at the main general hospital at each site. Interviews will seek to identify any changes to the crisis care pathway within the duration of the time series, including the introduction or withdrawal of services or new initiatives, including provision of services outside of the NHS mental health trust (e.g. by third sector agencies), changes in policy or protocol relating to the assessment and managing of psychiatric presentation to ED and seasonal impacts.

To account for potential confounding of any identified service reconfiguration or changes to models of care, sensitivity analysis comparing the months following the introduction of PDUs to the same period prior to the service change will be administered. These data will be used in interpreting time series curves for each of our outcomes (for example, where reconfiguration of community services was followed by a temporary spike in inpatient admissions, or where the introduction of a street triage service coincided with a sustained reduction in ED presentation).

## Analytical strategy

### Interrupted time series

We will use segmented regression analysis of interrupted time series data [34, 43] for the ITS. This approach minimizes threats to internal validity while maximizing external validity by taking into account any serial correlation, background variation and underlying trends independent of the intervention over time. Segmented regression analysis is the most common method used in ITS of healthcare interventions [44] and has previously been successfully used to predict emergency department (ED) presentations and hospital admission [45, 46], including psychiatric admissions and mental health-related ED visits [47].

Initially, for each outcome, to check for serial auto-correlation due to repeated measures, we will examine the plot of residuals from regression analyses and use the Durbin-Watson test [48]. Where no significant autocorrelation is detected, we will use a simple time series regression model. If significant autocorrelation exists, we will investigate several autocorrelation structures and adjust for effects as required. For example, where correlations due to seasonal associations are observed, monthly or seasonal dummy variables will be introduced in the model. Where weekly/monthly observations are not independent of one another, we will include a correlation structure based on the most appropriate autoregressive and moving average terms (assessed using plots of autocorrelation and partial correlation). Akaike’s and Schwarz’s Bayesian information criteria will be used to select most appropriate models.

For outcomes relying on count data (e.g., rate of inpatient admissions per 1000 trust catchment population), a generalized linear model for a Poisson distribution (or negative binomial model where count data is overdispersed) using a segmented approach will be fitted [34]. For each outcome, we will calculate regression coefficients corresponding to both change in level (outcome) and trend (slope) after the introduction of PDUs. In this model, the estimated parameters of interest are as follows: 1. the underlying trend prior to PDU introduction (b1); 2. the level change immediately following PDU introduction (b2); 3. the slope change from pre- to post-PDU introduction (using the interaction between time and change; b3); 4. the trend (slope change) following PDU introduction (b1_b3). External validity will be explored by examination of primary outcomes in subgroups, including, for example, gender. Graphical analysis will help to identify any stepwise change in outcome measures as a result of the introduction of PDUs and detect changes in activity patterns before and after the introduction of PDUs. We will conduct separate segmented regression analyses at each PDU site to provides individual estimates (and allow for evaluation of PDU implementation at a local level). Subsequently, to estimate overall effects, individual estimates of intervention effect will be pooled across sites (using inverse variance weights in a meta-analytical model to account for heterogeneity [49];).

### Synthetic control study

We will use the Generalised Synthetic Control (GSC) method [50] to compare the outcomes of patients attending a treated trust after the introduction of a PDU, to that of patients attending selected control trusts. The GSC method is in the spirit of a traditional synthetic control method in that it effectively re-weights the controls units to create a so-called ‘synthetic control’ that has similar values of the outcomes and covariates in the pre-intervention period. The idea is that this similarity is assumed to extend into the post-intervention period, providing an estimate of the outcomes that would have been expected at the trust if the PDU had not been introduced. These are referred to as the counterfactual outcomes.

The approach is as follows. For each treated trust, we first select a subset of the available control trusts that are as ‘similar’ as possible to the treated trust in the period prior to the introduction of the PDU. Similarity will be assessed in terms of variables such as attendances, deprivation, patient profile and location profile, e.g. by using the NHS TRUST Peer-Finder Tool [51]. The GSC method then uses the data from the selected control group, as well as the pre-intervention data from the treated trust, to estimate a synthetic control, and hence the counterfactual outcomes for the treated unit. The difference between these counterfactual outcomes and the observed outcomes in the treated unit in the period following the introduction of the PDU is assumed to provide estimates of the causal impact of the PDU on the outcomes in the treated unit. The precision of estimates will be assessed using parametric bootstrap procedures as described in [50] to generate *P*-values and confidence intervals.

Estimates will be risk-adjusted for variables that reflect changes over time in the characteristics of the denominator population, i.e. those who are able to have the outcome. For example, when looking at the rate of ED psychiatric presentations per 1000 trust catchment, the characteristics of the trust catchment population are relevant; when looking at the proportion of 4-h psychiatric ED breaches, the characteristics of psychiatric ED presentations are relevant. Estimates of overall effect will be made using the same methods as described for the ITS analysis.

### Sensitivity analysis to other changes on the crisis care pathway

To account for potential confounding of any identified service reconfiguration or changes to models of care, an ITS sensitivity analysis comparing the months following the introduction of PDUs to the same period prior to the identified service change will be administered. These data will be used in interpreting time series curves for each of our outcomes (for example, where reconfiguration of community services was followed by a temporary spike in inpatient admissions, or where the introduction of a street triage service coincided with a sustained reduction in presentations at the ED).

#### Coproduction and patient and public involvement

Four researchers on the team have lived experience of mental health issues, including the project manager and actively draw on and use this experience in their approach to and way of working in research. We have over 12 years’ experience of this approach as a research group and have an established culture which supports this way of working [52, 53]. In addition, service users outside of the research team have considerable input into the design, implementation and analysis of the research, including St George’s Peer Expertise in Education & Research (PEER) service user reference group and a Lived Experience Advisory Panel.

## Dissemination

We will disseminate the research widely using peer reviewed scientific journals, conference presentations and websites, including working with people with lived experience of mental health issues to ensure the results are available to a wide audience. No professional writers will be used.

## Discussion

Whilst the ITS method is suitable for assessing the impact of large organizational changes, it is important to plan how to address potential limitations. The biggest challenge to the study is from both national and local changes to the crisis care pathway (and service use) independent of the introduction of the PDUs during the study period. We will be constructing a detailed timeline of the crisis care pathway for each site, gathering data about any changes and the dates of these changes. Where the effect of the changes can be numerically estimated, the impact of these effects will be investigated using a sensitivity analysis.

The supplementary synthetic control study will control for national changes and trends over the 4 year period studied by contrasting outcomes in the treated trust with outcomes from trusts elsewhere in England with similar characteristics to the treated trust, but which do not have a PDU. This is achieved by creating a synthetic control that is assumed to provide an estimate of the hospital use that would have been expected at the treated trust in the absence of the introduction of the PDU. However, it is possible that the synthetic control is inappropriate, and not reflective of the treated trust before the PDU was introduced, nor of how it might have behaved afterwards. If this is the case, then our findings might be due to systematic differences between the treated trust and the synthetic control, rather than changes in how care was delivered. In the absence of a randomized controlled trial, it is not possible to eliminate this risk but the study is designed to reduce it as much as possible by using a control group with trusts that have similar characteristics to the treated trust, using risk adjustment to take account of any differences in the patients using hospital services over time and running additional sensitivity analyses to confirm that findings are robust to changes in the number of trusts in the control group and number of pre-intervention time periods used to determine the synthetic control.

Instrumentation [35], in which the measurement method changes during the intervention and evaluation period could also be an issue in the synthetic control study, in which we link between different datasets which cover different time periods. However, any changes in instrumentation will also be present for the synthetic control, so the change in instrumentation will balance between the two groups. The ITS design can be susceptible to fluctuating trends and cycles – we will statistically control for autocorrelation if indicated.

This study is well designed to identify changes to important service level measures of the functioning of the mental health crisis care pathway due to the PDU, allowing the trust-wide effects of PDUs to be estimated. The limitations of the ITS design are appropriately addressed by the inclusion of the synthetic control study, sensitivity analysis, and statistical methods selected. The selection of sites which have variation in PDU configuration will allow inferences to be made about the optimum design of decision units. It is possible that PDUs increase fragmentation in the crisis care pathway and do not have a beneficial effect on some or all service level measures. This study is well designed to identify such possibilities and will provide valuable information for Commissioners and trusts to address the pressure on the mental health crisis care system and will make an important contribution to the evaluation of PDUs and similar units in addressing challenges to mental health crisis care internationally.

## Data Availability

Not Applicable.

## Abbreviations

ED: Emergency Department; also known as Accident and Emergency
FOI: Freedom of Information
GSC: Generalised Synthetic Control
ITS: Interrupted Time Series
PDU: Psychiatric Decision Unit
PEER: Peer Expertise in Education & Research
